# Role of blood cultures during continuous renalreplacement theraphy in septic patients

**DOI:** 10.1186/2197-425X-3-S1-A59

**Published:** 2015-10-01

**Authors:** JA Monsalve- Naharro, P Cuesta-Montero, E Domingo-Chiva, S Plata-Paniagua, A López-Pérez, F Martinez-Lopez, JM Jimenez-Vizuete

**Affiliations:** Complejo Hospitalario Universitario de Albacete, Anesthesiology and Critical Care, Albacete, Spain; Complejo Hospitalario Universitario de Albacete, Pharmacy, Albacete, Spain; Complejo Hospitalario Universitario de Albacete, Albacete, Spain

## Introduction

Adequate microbiological diagnosis through blood cultures (BC) is essential to optimize the treatment of septic patients. However, their extraction in critically ill patients with continuous renal replacement therapy (CRRT) is poorly studied and characterized. CRRT can modify clinical signs of bacteriemia like fever and therefore the indication of obtaining BC. It is also unknown if the therapy itself can alter the results of BC. The routine of BC extraction every 24 or 48 hours is controversial and could be harmful.

## Objectives

To describe the results of blood cultures obtained during treatment with CRRT in septic patients with acute renal failure.

## Methods

Observational retrospective study of a cohort of septic patients admitted to a critical care unit with acute renal failure who required CRRT. BC were extracted routinly or by medical criteria. The study period ranged from May until September 2011.

The variables collected were: positivity rate, contamination rate, microbiological agent most frequently isolated and temporary positivity ratio (more or less than 7 days).

We considered the recomendations of the Spanish Society of Infectious Disease and Clinical Microbiology to defne the contamination or positive BC.

## Results

33 patients were included (57,6% males). Median of age was 66 years (IQR: 60-77). The diagnosis were: 23 septic shock (70%), 8 severe sepsis (25%) and 2 moderate sepsis (5%). The average score of APACHE II was 20,5. The mortality asociated was 40% (13 patients).148 patients undergoing CRRT cycles were collected. 104 BC were obtained from 66 of these CRRT cycles. Microbiological results were: negative in 78 BC (75%); 18 positive BC contamination (17,3%, coagulase negative Staphylococcus in all cases); 8 true positive BC (7,7%, corresponding to 5 patients).

For these 5 patients, BC were collected 7 days after admission. The microbiological agents obtained in true positive BC were: Acinetobacter Baumanii and Staphylococcus epidermidis both in 3 BC each one, and Pseudomonas Aeruginosa and Serratia marcencens in 1 BC each one.

## Conclusions

Rentability of BC in this cohort of critically ill patients with CRRT is low, and associated with more than seven days after admission. There was a high rate of contamination.

CRRT can alter BC results and modify their rentability. It is necessary to clarify the role of routine BC in patients with CRRTin the absence of signs of infection.
